# Supplementary material for: The Safety and Efficacy of Citrus aurantium (Bitter Orange) Extracts and p-Synephrine: A Systematic Review and Meta-Analysis
Source: Nutrients. 2022 Sep 28;14(19):4019. doi: 10.3390/nu14194019 (PMC9572433; doi:10.3390/nu14194019)
Supplement: Supplementary file 1 [file nutrients-14-04019-s001.zip › nutrients-1937045-supplementary.pdf]

Supplementary materials

Table S1. Unauthorized synephrine (above maximal limit) products in the European Union in the RASFF from 1988-2019.

Year	Synephrine	Notified by
1988–2006	0	NA [1]
2007	3	Norway; Denmark (2)
2008	0	NA [1]
2009	1	Poland
2010	1	Poland
2011	1	France
2012	1	Sweden
2013	15	Finland (9); Denmark (6)
2014	5	Poland (2); Sweden (1); Germany (2)
2015	1	Norway
2016	16	Poland (1); Germany (9); Sweden (2); Netherlands (4)
2017	4	Germany (1), Sweden (3)
2018	4	Germany (1), Sweden (3)
2019	1	Sweden

[1] NA is abbreviation for “Not applicable”

Table S2. Characteristics of the trials.

Author and year	Synephrine form[1]	Study drug	Dosage	Design
Bui 2006	p-	54 mg	2 capsules daily	prospective, crossover, repeated-measure
Bush 2018	p-	103 mg	once, daily two capsules	within subjects
Colker 1999	p-	54 mg	once daily	parallel
Guiterrez-Hellín 2016	p-	3 mg/kg (average of 214 mg)	once daily	counterbalanced
Guiterrez-Hellín 2018	p-	3 mg/kg (average of 203 mg)	once daily	randomized
Guiterrez-Hellín 2020	p-	3 mg/kg (average of 213 mg)	once daily	randomized
Hoffman 2009	4-HMP	20 mg	3 capsules daily	crossover
Jung 2017a	p-	20 mg	1 serving daily	counterbalanced and crossover manner
Jung 2017b	p-	20 mg	1 foil packet per day approximately 15–30 min prior to exercise	parallel, prospective cohort
Kaats 2013	p-	24.4 (49 mg daily)	twice daily	parallel
Kalman 2000	p-	5 mg	twice daily	parallel
Min 2005	p- or m-	27 mg	1 tablet dose of bitter-orange dried-fruit extract	crossover
Sale 2006	p-	3 mg (6mg)	2 capsules daily	counterbalanced
Seifert 2011	p-	13 mg (total of 52 mg)	4 capsules	crossover
Shara 2016	p-	49-mg	1 single capsule daily	crossover
Stohs 2011	p-	50 mg	1 ounce of juice daily	parallel
Ratamess 2018	p-	103 mg	once daily two capsules	within subjects
Benjamim 2022	p-	180 mg	once 180 mg synephrine containing capsule	crossover

[1] *p-* stands for *para-* synephrine; *m-* is for *meta*-synephrine (phenylephrine) and 4-HMP means methyl-synephrine HCl

Table S3. Demography of patients.

Author and year	Patients	Average age (years)	Patient randomized	Overall dropout	Control	Intervention	Male, female
Bui 2006	healthy	26 ± 2	15	2	13	13	10 male, 5 female
Bush 2018	healthy and physically active	20.3 ± 1.3	16	2	8	8	10 male, 3 female
Colker 1999	healthy BMI > 25 kg/m2	>21	23	3	7	9	NK
Guiterrez-Hellín 2016	healthy and physically active	26.0 ± 7.2	18	0	9	9	NK
Guiterrez-Hellín 2018	healthy and physically active	25.0 ± 7.0	13	0	13	13	11 male, 2 female
Guiterrez-Hellín 2020	healthy	31 ± 6.9	14	1	13	13	11 male, 2 female
Hoffman 2009	healthy, 71.5 ± 17.2 kg; 17.3 ± 2.6% body fat	20.2 ± 1.2	10	0	10	10	5 male, 5 female
Jung 2017a	healthy (recreationally active)	22 ± 3	26	1	25	25	20 male 5 female
Jung 2017b	healthy, (resistance-trained male)	20.9 ± 3.9	80	0	27	26	80 male
Kaats 2013	healthy	51.3	75	8	25	25	15 male, 60 female
Kalman 2000	overweight adults, (body mass index >27 kg/m2)	42.07-43, 06	30	5	13	12	23 male, 7 female
Min 2005	healthy	24.9 ± 4.4	18	1	18	18	NK
Sale 2006	overweight, sedentary males	27±7	10	1	10	10	10 male
Seifert 2011	mildly overweight individuals Three subjects were pre-existing hypertensives (systolic blood pressure > 140 mmHg)	24.7 +7.4	23	0	11	12	9 male, 14 female
Shara 2016	healthy	25	18	0	18	18	9 male, 9 female,
Stohs 2011	healthy	NK	50	0	10	10	NK
Ratamess 2018	healthy	20.3 ± 1.3	16	0	8	8	13 male, 3 female
Benjamim 2022	healthy, physically active	18-30	17	5	12	12	17 male

[1] NA is abbreviation for "Not applicable"

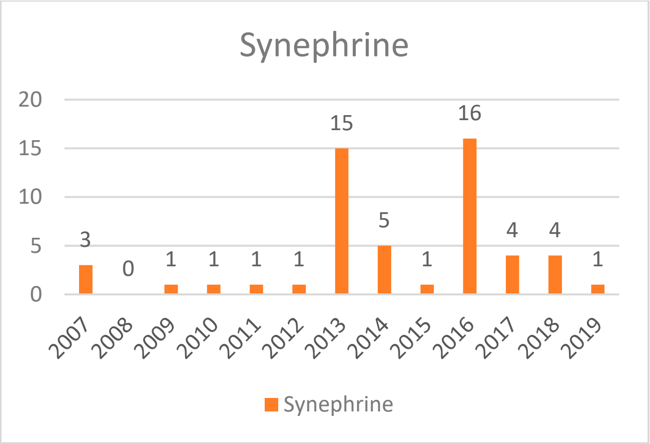

Figure S1. Unauthorized synephrine (above maximal limit) products in the RASFF from 2007-2019 (accessed on 31 Dec 2019).

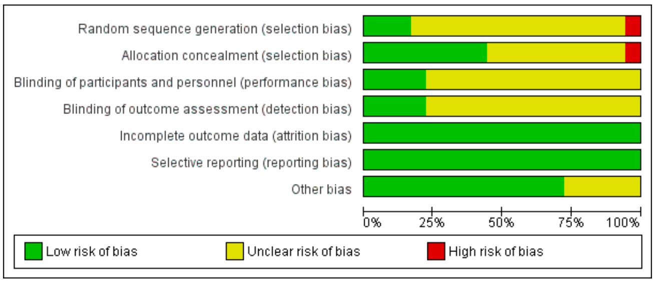

Figure S2. Risk of bias graph on synephrine meta-analysis.

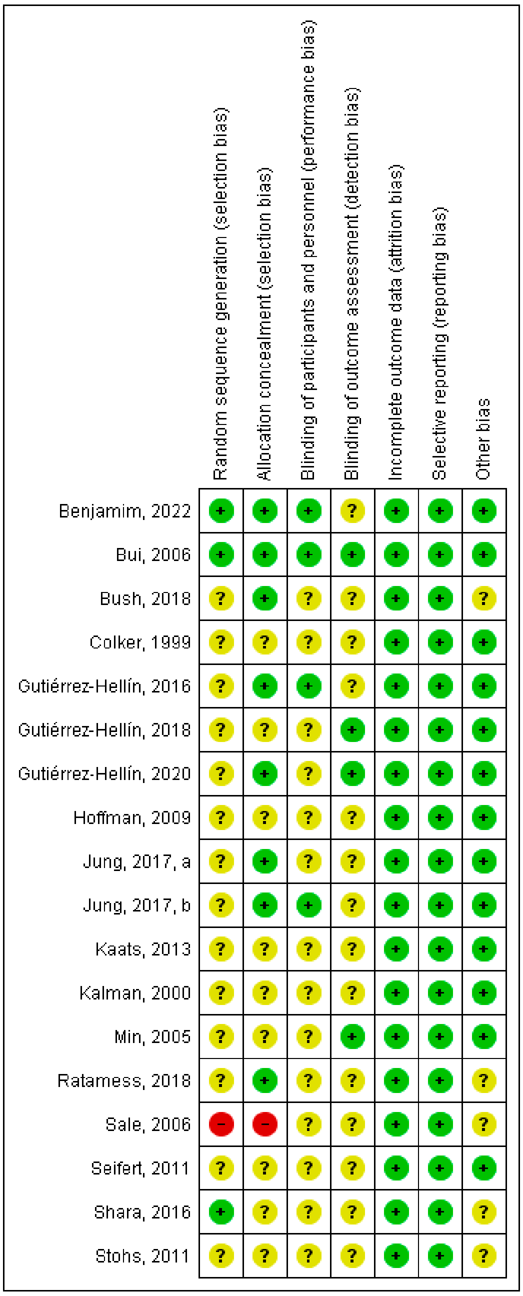

Figure S3. Risk of bias summary on synephrine meta-analysis.

Study or Subgroup	Synephrine Mean	Synephrine SD	Synephrine Total	Control Mean	Control SD	Control Total	Weight	Mean Difference IV, Random, 95% CI
Bush_3hr 2018	47.5	4.5	8	46	15.3	8	24.8%	1.50 [-9.55, 12.55]
Jung_2hr 2017a	113.8	15	25	101.5	14.4	25	32.7%	12.30 [4.15, 20.45]
Sale_2hr 2006	92	5.9	10	91.1	5.8	10	42.5%	0.90 [-4.23, 6.03]
**Total (95% CI)**			**43**			**43**	**100.0%**	**4.78 [-2.79, 12.35]**

Heterogeneity: Tau² = 28.29; Chi² = 5.57, df = 2 (P = 0.06); I² = 64%
Test for overall effect: Z = 1.24 (P = 0.22)

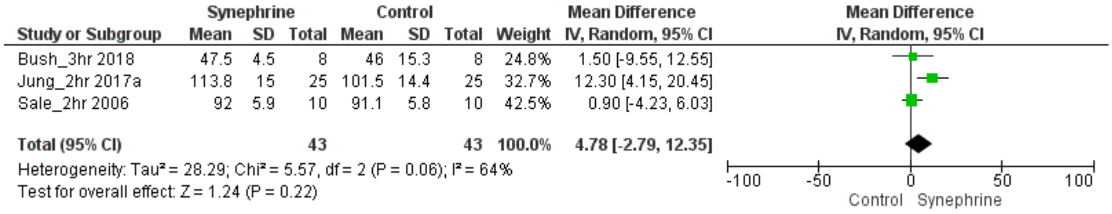

(**h/2**)

Figure S4. Forest plot diagram of synephrine on (**h/2**) blood glucose at short duration at intervention and control groups with one change of the time duration.

Study or Subgroup	Synephrine Mean	Synephrine SD	Synephrine Total	Control Mean	Control SD	Control Total	Weight	Mean Difference IV, Fixed, 95% CI
Hoffman 2009	0.86	0.05	10	0.87	0.09	10	19.6%	-0.01 [-0.07, 0.05]
Sale 2006	0.842	0.042	10	0.814	0.051	10	47.7%	0.03 [-0.01, 0.07]
Seifert 2011	0.83	0.07	12	0.86	0.05	11	32.7%	-0.03 [-0.08, 0.02]
**Total (95% CI)**			**32**			**31**	**100.0%**	**0.00 [-0.03, 0.03]**

Heterogeneity: Chi² = 3.29, df = 2 (P = 0.19); I² = 39%
Test for overall effect: Z = 0.11 (P = 0.91)

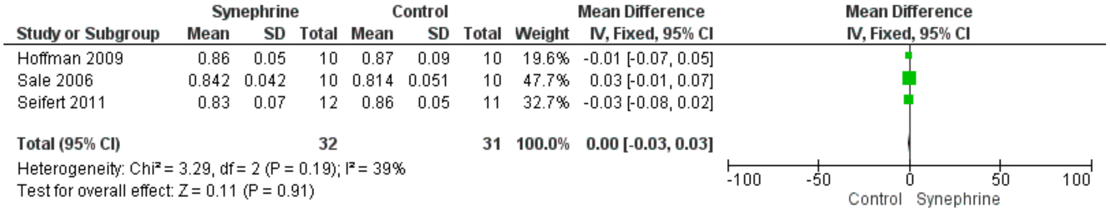

Study or Subgroup	Synephrine Mean	Synephrine SD	Synephrine Total	Control Mean	Control SD	Control Total	Weight	Mean Difference IV, Fixed, 95% CI
Hoffman 2009	0.86	0.05	10	0.87	0.09	10	17.7%	-0.01 [-0.07, 0.05]
Sale_2hr 2006	0.823	0.045	10	0.789	0.039	10	52.8%	0.03 [-0.00, 0.07]
Seifert 2011	0.83	0.07	12	0.86	0.05	11	29.5%	-0.03 [-0.08, 0.02]
**Total (95% CI)**			**32**			**31**	**100.0%**	**0.01 [-0.02, 0.03]**

Heterogeneity: Chi² = 4.48, df = 2 (P = 0.11); I² = 55%
Test for overall effect: Z = 0.54 (P = 0.59)

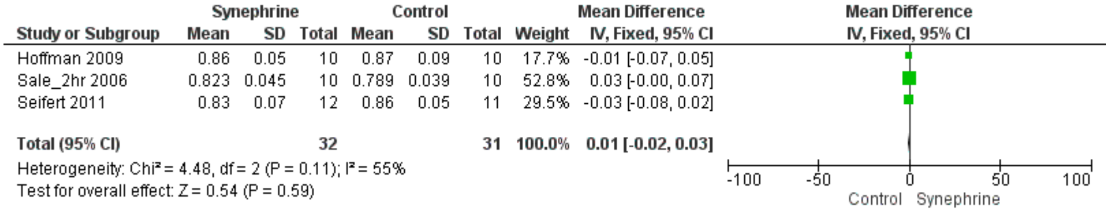

Study or Subgroup	Synephrine Mean	Synephrine SD	Synephrine Total	Control Mean	Control SD	Control Total	Weight	Mean Difference IV, Random, 95% CI
Hoffman 2009	0.8	0.04	10	0.87	0.07	10	48.6%	-0.07 [-0.12, -0.02]
Sale 2006	0.823	0.045	10	0.789	0.039	10	51.4%	0.03 [-0.00, 0.07]
**Total (95% CI)**			**20**			**20**	**100.0%**	**-0.02 [-0.12, 0.09]**

Heterogeneity: Tau² = 0.00; Chi² = 10.77, df = 1 (P = 0.001); I² = 91%
Test for overall effect: Z = 0.32 (P = 0.75)

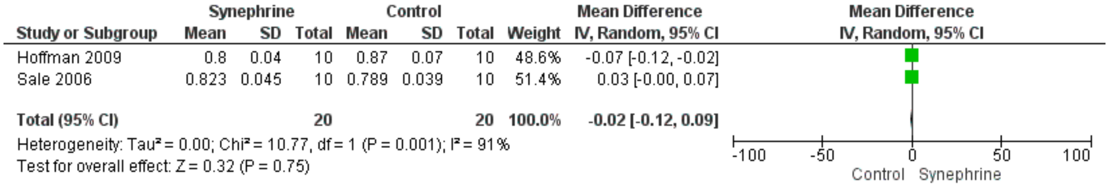

Study or Subgroup	Synephrine Mean	Synephrine SD	Synephrine Total	Control Mean	Control SD	Control Total	Weight	Mean Difference IV, Random, 95% CI
Hoffman 2009	0.79	0.04	10	0.86	0.07	10	48.1%	-0.07 [-0.12, -0.02]
Sale 2006	0.799	0.024	10	0.768	0.045	10	51.9%	0.03 [-0.00, 0.06]
**Total (95% CI)**			**20**			**20**	**100.0%**	**-0.02 [-0.12, 0.08]**

Heterogeneity: Tau² = 0.00; Chi² = 11.21, df = 1 (P = 0.0008); I² = 91%
Test for overall effect: Z = 0.35 (P = 0.73)

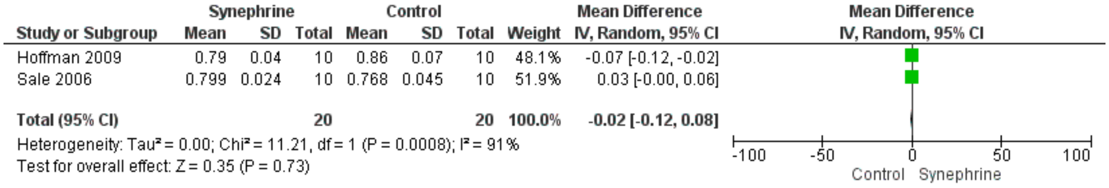

(**i**)

Figure S5. Forest plot diagram of synephrine on (**i**) RER values after 1-3 hours (1 hour two times with one change in time interval) at intervention and control groups.
